# Gene Body Methylation in Plants: Mechanisms, Functions, and Important Implications for Understanding Evolutionary Processes

**DOI:** 10.1093/gbe/evac038

**Published:** 2022-03-17

**Authors:** Aline M Muyle, Danelle K Seymour, Yuanda Lv, Bruno Huettel, Brandon S Gaut

**Affiliations:** 1 Ecology and Evolutionary Biology, University of California, Irvine, USA; 2 Laboratoire ‘Biométrie et Biologie Evolutive’, CNRS/Université Lyon 1, Lyon, France; 3 Botany & Plant Sciences, University of California, Riverside, USA; 4 Provincial Key Laboratory of Agrobiology, Institute of Crop Germplasm and Biotechnology, Jiangsu Academy of Agricultural Sciences, Nanjing, China; 5 Max Planck Genome Centre Cologne, Max Planck Institute for Plant Breeding, Cologne, Germany

**Keywords:** epigenetics, gene expression, transcription, DNA methylation, population epigenomics

## Abstract

Gene body methylation (gbM) is an epigenetic mark where gene exons are methylated in the CG context only, as opposed to CHG and CHH contexts (where H stands for A, C, or T). CG methylation is transmitted transgenerationally in plants, opening the possibility that gbM may be shaped by adaptation. This presupposes, however, that gbM has a function that affects phenotype, which has been a topic of debate in the literature. Here, we review our current knowledge of gbM in plants. We start by presenting the well-elucidated mechanisms of plant gbM establishment and maintenance. We then review more controversial topics: the evolution of gbM and the potential selective pressures that act on it. Finally, we discuss the potential functions of gbM that may affect organismal phenotypes: gene expression stabilization and upregulation, inhibition of aberrant transcription (reverse and internal), prevention of aberrant intron retention, and protection against TE insertions. To bolster the review of these topics, we include novel analyses to assess the effect of gbM on transcripts. Overall, a growing body of literature finds that gbM correlates with levels and patterns of gene expression. It is not clear, however, if this is a causal relationship. Altogether, functional work suggests that the effects of gbM, if any, must be relatively small, but there is nonetheless evidence that it is shaped by natural selection. We conclude by discussing the potential adaptive character of gbM and its implications for an updated view of the mechanisms of adaptation in plants.

**Significance**

Gene body methylation (hereafter gbM) is a common phenomenon in plants and can affect up to 60% of genes in some species. It has been controversial whether gbM has any function in plants, but recent findings suggest it is under selection and correlated with fitness. Here, we review the scientific literature, include novel analyses, and discuss the potential role of gbM in rapid evolutionary change.

## Introduction

Epigenetics is the study of changes in gene expression that can be inherited through cell divisions (either mitotic or meiotic) that are not due to modifications in the DNA sequence (Holliday 1994; Cavalli and Heard 2019). A longstanding question is whether epigenetics can play a role in adaptation (Charlesworth et al. 2017; Cavalli and Heard 2019; Boquete et al. 2021). Cavalli and Heard (2019) stated that “a direct demonstration that other molecules, in addition to DNA [sequence], carry substantial heritable information would represent an important conceptual change in evolutionary biology.” Theoretically, epigenetic marks may be the basis for this conceptual change. If epigenetic marks affect fitness, if they are inherited through generations, and if they epimutate over time, then they can be the target of selection and facilitate adaptation.

Cytosine methylation is one common epigenetic mark that is generally found in eukaryotes, including vertebrates, insects, fungi, and plants (Zemach and Zilberman 2010; Schmitz et al. 2019). In some of these groups, cytosines are methylated only in a single context when they are part of a CG dinucleotide. In plants, however, cytosine methylation occurs in three sequence contexts—CG, CHG, and CHH (where H stands for A, T, or C). Methylation marks in these three contexts are produced by different biochemical pathways and have different patterns of inheritance. For example, epimutation accumulation lines in *Arabidopsis thaliana* have demonstrated that genome-wide methylation divergence at CG dinucleotides increases throughout >30 generations (van der Graaf et al. 2015), illustrating that plant CG DNA methylation is transmitted from generation to generation and epimutates over time (Yao et al. 2021). In contrast, CHH methylation is mostly erased by demethylation in the *A. thaliana* male germline and later reset during embryonic development (Calarco et al. 2012). Therefore, CHH methylation is only transmitted partially over, at most, one or a few generations (with the interesting exception of some asexual plants without meiosis; Boquete et al. 2021). The transgenerational inheritance of the third context—CHG methylation—remains unclear. Although CHG methylation is retained during gametogenesis (Calarco et al. 2012), epimutation accumulation lines in *A. thaliana* do not diverge for CHG methylation over generations (van der Graaf et al. 2015), suggesting that CHG methylation is not inherited at a genome-wide scale. It is possible, however, that some genomic sites inherit CHG methylation over a few generations, especially in some asexual species (Boquete et al. 2021). To summarize, of the three methylation contexts in plants, methylation in CG dinucleotides is most prone to transgenerational inheritance and is therefore the best candidate for epigenetic adaptation.

To consider the possibility of epigenetic adaptation, it is also important to know where these marks reside in the genome. In flowering plants, patterns of DNA methylation vary among genomic regions. Methylation in all three contexts silences transposable elements (TEs) and prevents activity at regulatory elements (Luo et al. 2018; Schmitz et al. 2019). Both CHG and CHH genic methylation types are associated with reduced expression levels, as is CG methylation in promoter regions (Zhang et al. 2006; Niederhuth et al. 2016). In contrast, the exons of some genes (∼20% of *A. thaliana* genes; Takuno and Gaut 2012) are methylated only in the CG context, a phenomenon called gene body methylation (hereafter gbM). gbM is mostly found in moderately and constitutively expressed housekeeping genes (Zhang et al. 2006; Neri et al. 2017; Schmitz et al. 2019). However, since its initial discovery, the topic of gbM function has been controversial (Zhang et al. 2006; Teixeira and Colot 2009; Bewick et al. 2017; Zilberman 2017). If it has no function, it is obviously unlikely to contribute to adaptive processes.

Here, we review our current knowledge of gbM in plants, with the ultimate goal to critically evaluate whether it has a function and may be a target for natural selection. We start by presenting the mechanisms of plant gbM establishment and maintenance because these mechanisms are crucial for understanding how selection could act on this epigenetic state. We then consider the evolution of gbM, specifically whether gbM is a neutral manifestation of epigenomic dynamics or whether there is evidence that it can be advantageous. Adaptive arguments presume that gbM has a phenotype on which natural selection can act. Some, but certainly not all, recent work has established a connection between gbM and gene expression, but questions about generality and mechanisms remain. To address these questions, we review functional analyses of mutants and also comparative epigenomic approaches that have studied hypothetical functions of gbM, namely, its potential role in regulating and stabilizing expression, preventing aberrant transcription, and improving the fidelity of intron splicing. Finally, we present a model synthesizing the prevalence, distribution, and effect of gbM with its potential evolutionary significance.

## GbM Establishment and Maintenance Mechanisms

CG methylation is maintained during plant cell division by Methyltransferase 1 (MET1), which adds a methyl group on the symmetrical CG dinucleotide of a complementary DNA strand (Kawashima and Berger 2014). Epimutation accumulation lines in *A. thaliana* have shown that the maintenance of CG methylation by MET1 is an inherently error-prone process, with the epimutation rate estimated to be ∼10^−3^ per generation per haploid epigenome for the loss of CG methylation in genes (van der Graaf et al. 2015). Without methylation maintenance mechanisms, CG methylation is quickly diluted and lost over cell divisions, as demonstrated by the absence of CG methylation in *A. thaliana met1* mutants (Cokus et al. 2008).

Studies on *Eutrema salsugineum*, a close relative of *A. thaliana*, have recently clarified the mechanisms responsible for the establishment of gbM in plants (Bewick et al. 2016). *Eutrema salsugineum* lacks both gbM and the *Chromomethylase 3* (*CMT3*) gene. The link between *CMT3* loss and the absence of gbM was at first enigmatic because, until recently, CMT3 was not known to methylate CG sites. It is now known that the CMT3 protein is involved in a self-reinforcing feedback loop: CMT3 recognizes the histone mark H3K9me2 (histone H3 lysine 9 dimethylation) and then de novo methylates nearby cytosines predominantly in the CHG context but also occasionally in the CG context. CHG DNA methylation in turn leads to H3K9 methylation by SU(VAR) Homologue 4 (SUVH4), leading to a positive feedback loop between CHG methylation and H3K9 methylation (Johnson et al. 2007). CHG methylation typically suppresses transcription; however, in *A. thaliana*, CHG methylation is removed in transcribed genes due to active demethylation of H3K9 by *Increased in Bonsai Methylation 1 (IBM1)* (Saze et al. 2008; Miura et al. 2009).

The joint loss of *CMT3* and gbM evolved independently in two Brassicaceae species, corroborating their association. However, *cmt3* mutants in *A. thaliana* have shown that CMT3 does not affect the maintenance of gbM once it is established (Stroud et al. 2013), suggesting the action of CMT3 is limited to gbM establishment (Bewick et al. 2016; Niederhuth et al. 2016). Interestingly, transgenic reinsertion of *CMT3* into *E. salsugineum* re-established genic methylation in all three contexts in a subset of genes (Wendte et al. 2019). This subset of genes has been called “CHG-gain” genes, and these genes tend to be orthologous to gbM genes in *A. thaliana* (Wendte et al. 2019). After the *CMT3* transgene was lost, CHG-gain genes only maintained methylation in the CG context, presumably due to maintenance by MET1 (Wendte et al. 2019). On average, CHG-gain genes are longer, contain more exons, and exhibit a moderate—but on average higher—level of expression than non-CHG-gain genes (Wendte et al. 2019). CHG-gain genes are also enriched for CWG trinucleotides (CAG and CTG) as opposed to CCG trinucleotides, consistent with the preferred substrate of CMT3 (Gouil and Baulcombe 2016; Stoddard et al. 2019). Finally, CHG-gain genes have a higher frequency of CHG cytosines compared to non-CHG-gain genes (Wendte et al. 2019).

**Fig. 1.**
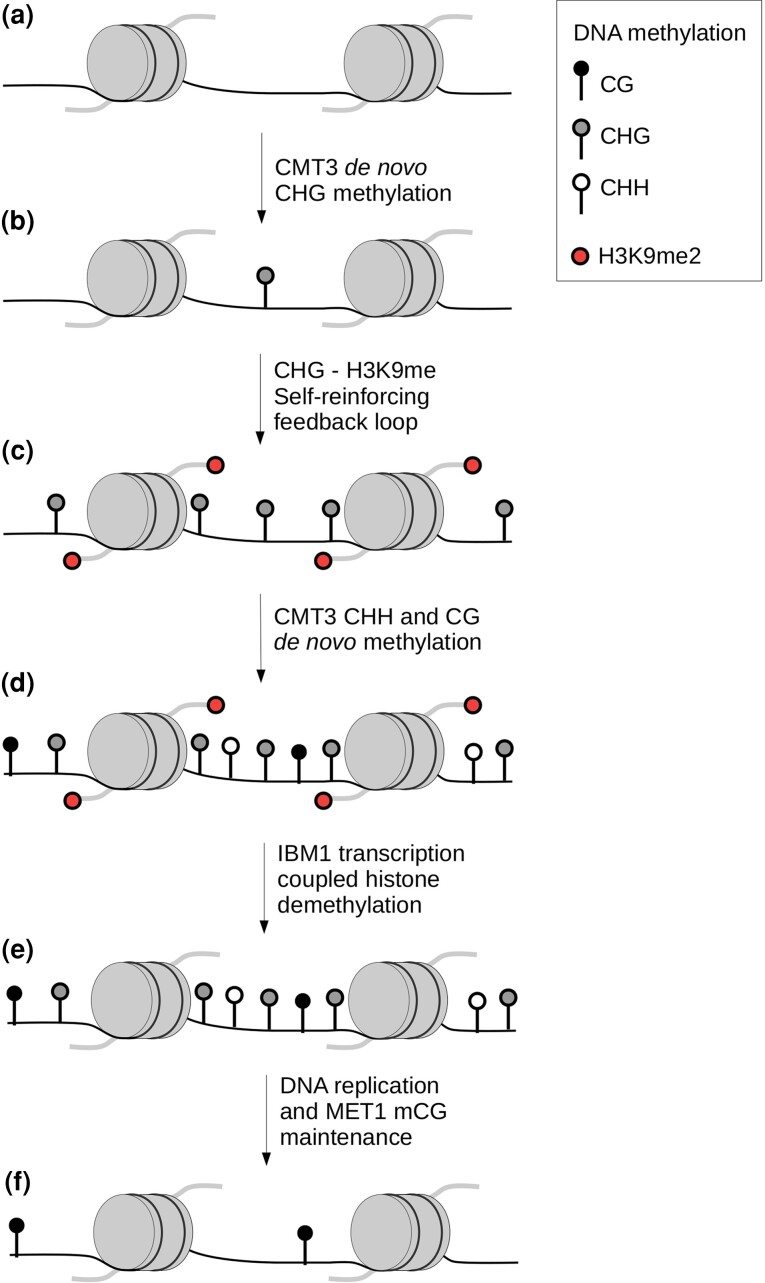
The establishment and maintenance of gbM in plants. The DNA is represented as a line coiled around nucleosomes. Red dots indicate methylated H3K9 tails. CG, CHG, and CHH DNA methylation are drawn as black, gray, and white lollipops, respectively. (*a* and *b*) CMT3 induces de novo methylation at CHG sites of genes associated with inaccessible chromatin marks and heterochromatin histone variants (Papareddy et al. 2021). (*b* and *c*) CHG-H3K9me2 self-reinforcing feedback loop is then established. (*c* and *d*) CMT3 preferentially de novo methylates CWG sites but to a lesser extent also methylates other contexts, such as CG. (*d* and *e*) Demethylation of H3K9 by IBM1 is coupled to gene transcription. (*f*) After a few cell divisions, only CG methylation (mCG) remains due to MET1 maintenance.

The role of CMT3 in genic de novo methylation was recently confirmed in *A. thaliana* mutants that hyperexpress *CMT3* during late embryonic development (Papareddy et al. 2021). CMT3 hyperexpression induces embryonic hypermethylation predominantly in the CWG context, but hypermethylation is also found in other contexts, including CG dinucleotides. These findings confirm that CMT3 is sloppy and can methylate contexts other than CHG. Methylation changes caused by embryonic CMT3 hyperexpression were maintained over cell divisions and still observed in 3-week-old plants, consistent with the model that CMT3-induced epimutations give rise to gbM that can be maintained by MET1 across cell divisions and generations. The same gene patterns were repeatedly observed in independent transgenic lines, confirming that CMT3 hypermethylation is not stochastic and tends to target a specific gene set (Papareddy et al. 2021). CMT3-induced hypermethylation was also enriched in genes characterized by inaccessible chromatin marks and heterochromatin histone variants (Papareddy et al. 2021). Altogether, these observations lead to a model in which gbM establishment is caused by the recruitment of CMT3, the formation of a feedback loop that ultimately produces CHG, CHH, and CG methylation, the eventual removal of CHG and CHH methylation, and the maintenance of the remaining CG methylation by MET1 (fig. 1).

## gbM Gene Characteristics and Evolution

gbM genes are typically defined statistically as being significantly more methylated than the genic average in the CG context and significantly less methylated than the genic average in the CHG and CHH contexts (Takuno and Gaut 2012). Once defined, the proportion of gbM genes varies greatly across species, with as many as ∼60% of genes in *Mimulus guttatus* but 0% in *Marchantia polymorpha*, *Physcomitrella patens,* and *E. salsugineum* (Niederhuth et al. 2016; Takuno et al. 2016; Niederhuth and Schmitz 2017). The lack of gbM in a few species has been used to argue that gbM is dispensable and thus has no function (Bewick et al. 2016, 2019). However, the loss of gbM in a few species does not imply that it is nonfunctional in all plants (Zilberman 2017).

A remarkable feature of gbM is that it is enriched over a conserved set of orthologs among species as distantly related as ferns and angiosperms (Takuno and Gaut 2013; Seymour et al. 2014; Niederhuth et al. 2016; Takuno et al. 2016; Seymour and Gaut 2020). Two alternative hypotheses can explain the remarkable conservation of gbM. The first is the biased establishment of gbM in a subset of specific genes with inaccessible chromatin marks and heterochromatic (H3K9me2) histone variants (Wendte et al. 2019). If these biases are conserved across species, they could explain the distribution of gbM across both genes and species. This first hypothesis is neutral with respect to selection because it does not assume that gbM has any effect on fitness. Instead, in this scenario, gbM is a consequence of CMT3 activity that is retained and transmitted over generations by MET1 (Teixeira and Colot 2009; Wendte et al. 2019; Papareddy et al. 2021).

An observation in favor of the neutral hypothesis is that gbM genes share many characteristics of the CHG-gain genes described previously. That is, the genes targeted by CMT3 are like gbM genes, in that they are generally characterized as being constitutively expressed at moderate levels and tend to be longer than unmethylated genes, with more exons and a higher frequency of CAG and CTG (as opposed to CCG) trinucleotides (Zhang et al. 2006; Lister et al. 2008; Takuno and Gaut 2012, 2013; Bewick et al. 2016, 2017; Niederhuth et al. 2016; Takuno et al. 2016). Moreover, CHG-gain genes in *E. salsugineum* tend to be orthologous to gbM genes in *A. thaliana* (Wendte et al. 2019). This observation suggests gbM establishment is biased toward specific genes, potentially explaining the conservation of gbM between orthologs (Wendte et al. 2019; Papareddy et al. 2021). The overlap between CHG-gain and gbM is not complete because ∼40% of CHG-gain genes in *E. salsugineum* are orthologous to a gbM gene in *A. thaliana* (Wendte et al. 2019). One explanation for the imperfect overlap between CHG-gain genes and gbM genes is that they were defined in different species – that is, CHG-gain genes were defined in *E. salsugineum* and gbM genes in *A. thaliana.* Moreover, these two species diverged 47 Ma (Arias et al. 2014), which may be ample time for the targets of CMT3 to diverge. Finally, another plausible explanation is temporal. The CHG-gain genes in *E. salsugineum* were established experimentally over only a few generations; the continuation of this experiment over a much longer timeframe could lead to the establishment of methylation within more genes, potentially increasing the 40% overlap.

An alternative hypothesis is that the conservation of gbM across genes and species is shaped in part by the action of natural selection (Zilberman 2017). Under this scenario, specific subsets of genes are targeted for de novo establishment of gbM, but selection on or against gbM removes or maintains CG methylation in different gene sets. At least three observations support the hypothesis that some gbM is under selection. First, DNA methylation is mutagenic and elevates C to T substitutions (Bird 1980). Therefore, the conservation of gbM in a specific set of orthologous genes is surprising, especially because gbM genes are generally enriched for housekeeping and other important functions and evolve more slowly than unmethylated genes (Takuno and Gaut 2012, 2013; Takuno et al. 2017; Seymour and Gaut 2020). This suggests the possibility that the mutagenic nature of methylation is compensated by an advantageous effect that maintains gbM in specific genes (Zilberman 2017). The second observation in favor of the selective hypothesis comes from the comparison of the gbM status in orthologous genes of eight grass species, where shifts in the gbM status of genes are almost exclusive to the tips of the phylogeny (i.e., in a single species) (Seymour and Gaut 2020). This pattern suggests that shifts in gbM are deleterious and generally not favored over evolutionary time (table 1); however, it is also possible that the pattern is driven by epimutational biases.

**Table 1.** Review of the Literature on the Possible Functions of gbM and Selective Pressures That Act on It

Reference	Species and genotypes	Data type	gbM upregulates gene expression	gbM stabilizes gene expression	gbM prevents internal transcription start	gbM prevents aberrant transcription termination	gbM prevents antisens transcription	gbM prevents intron retention	gbM prevents TE insertion	gbM is under selection
Regulski et al. (2013)	Maize B73	BS-seq							Yes (Mu element)	
Bewick et al. (2016)	*Eutrema salsugineum* WT, *Arabidopsis thaliana* WT + *met1* epiRIL + *met1, sdg7, sdg8*	BS-seq, RNA-seq	No				No	No		
Vidalis et al. (2016)	92 WT *A. thaliana*	BS-seq								No
Meng et al. (2016)	135 *A. thaliana* wild accessions	BS-seq, RNA-seq	Yes (for a few genes)							
Takuno et al. (2017)	*A. thaliana*, *A. lyrata*	BS-seq, RNA-seq	Trend	Yes						
Steige et al. (2017)	*Capsella grandiflora*	BS-seq, RNA-seq		Yes						
Neri et al. (2017)	Mouse embryonic stem cells WT + *Dnmt3b* + *SetD2* knockdown + DNMT3B rescue	Dnmt3b ChIP-seq, BS-seq, RNA-seq, Pol II ChIP-seq, CAPIP-seq, DECAP-seq			Yes					
Teissandier and Bourc’his (2017)	Mouse embryonic stem cells WT + *Dnmt- tKO* (triple mutant) + chemical inhibition of methylation	RNA-seq			No					
Muyle and Gaut (2019)	*E. salsugineum*, *A. thaliana*	BS-seq, RNA-seq	Yes							
Bewick et al. (2019)	*E. salsugineum*, *A. thaliana* WT + *met1* epiRIL	BS-seq, RNA-seq	No							
Horvath et al. (2019)	*A. thaliana* WT	BS-seq, Single-cell RNA-seq		Yes				Yes		
Seymour and Gaut (2020)	8 species of the grass family (Poaceae)	BS-seq, RNA-seq	Yes	Yes						Yes
Choi et al. (2020)	*A. thaliana* WT + *met1* + *h1* + *h1, met1*	BS-seq, RNA-seq, decap-seq					Yes, jointly with histone H1			
Muyle et al. (2021)	1,001 WT *A. thaliana*	BS-seq, RNA-seq	Yes	Yes						Yes
Shahzad et al. (2021)	1,001 WT *A. thaliana*, *A. thaliana* mutant collection	BS-seq, RNA-seq	Yes							
Li et al. (2021)	WT *A. thaliana* and *met1* mutant.	BS-seq, ONT DRS			Yes	Yes		Yes		
This manuscript	Maize WT, *A. thaliana* WT + *met1* *+* *met1, sdg7, sdg8*	BS-seq, RNA-seq, Isoseq			Yes in WT *A. thaliana* Isoseq data but not in maize nor in RNA-seq data		Yes in WT *A. thaliana* Isoseq data but not in maize	No		

Note.—Many studies contradict one another, suggesting that if gbM indeed has a function, its effect must be subtle.

The third observation is based on population genetic analyses because selection acting on gbM can be explicitly measured using DNA methylation variation among natural populations of a species. Indeed, if gbM is advantageous within a given gene, an unmethylated allele will be disadvantageous and removed by selection. In such a gene, only a small proportion of individuals should be observed with an unmethylated allele. To infer the intensity of selection, Charlesworth and Jain (2014) constructed a population model that relies on the site frequency spectrum (SFS) of epigenetic states. In an inspired analysis, Vidalis et al. (2016) applied this model to the SFS of CG sites within all genes of a sample of 92 *A. thaliana* individuals. They did not detect a deviation from neutrality (table 1), but this result came with two important caveats. The first is that the test is unlikely to be powerful with a small sample, particularly if gbM has a small impact on fitness. Vidalis et al. (2016) used the sample of 92 individuals that was available at the time, but larger samples now exist. The second is that CG methylation within genes is not limited to gbM genes but can also be found in genes that are methylated in all three contexts (i.e., TE-like methylation; Kawakatsu et al. 2016). Methylation in all three contexts within a gene can be caused by a nearby TE insertion, is known to suppress expression, and may be an indication of pseudogenization. Therefore, most genes with TE-like methylation are likely to be under different evolutionary pressures than gbM genes, such that analyzing both gbM and TE-like methylated genes together, as done by Vidalis et al. (2016), is likely to confound opposing selection pressures.

For all these reasons, we recently repeated the analyses of Vidalis et al. (2016) with the important difference that we separated gbM gene sets from any genes with TE-like methylation (Muyle et al. 2021). We also relied on larger data sets—that is, two distinct subsets of 876 and 120 individuals that originated from different sources—from the 1001 methylomes project in *A. thaliana* (Kawakatsu et al. 2016). To assess whether selection acts on the gbM state, we characterized the population frequency of methylation at the gene level to estimate the SFS of gene allelic states. Using the population genetic model of Charlesworth and Jain (2014), we inferred that genes with ancestral gbM in Brassicaceae were under significant selection to remain CG methylated in *A. thaliana* (table 1; Muyle et al. 2021) based on the larger data set. Conversely, ancestrally unmethylated genes in Brassicaceae were under selection to remain unmethylated in *A. thaliana*. We repeated the analyses on the smaller data set and also on an SFS drawn at the level of individual cytosines. The former had similar trends as the larger data set but without a significant effect of gbM, and the latter corroborated our gene-level analyses. That is, the overall impression is that CG sites within ancestrally gbM genes in Brassicaceae have been under selection to remain methylated in *A. thaliana*, while CG sites within ancestrally unmethylated genes have been under selection to be unmethylated in *A. thaliana* (Muyle et al. 2021). Importantly, the results were also confirmed after splitting the gene sets into CHG-gain and non-CHG-gain genes, as characterized by Wendte et al. (2019), showing that biases in epimutation rates between gene sets were not completely responsible for the inferred selection acting on gbM. In other words, this control using CHG-gain genes shows that *cis* effects (either genetic or epigenetic) that locally influence epimutation rates do not explain the inferred selective pressures.

Like all evolutionary analyses, there are caveats to this analysis, too. First, it relies on a model that simplifies the evolutionary process and includes assumptions that do not strictly fit the study organism (e.g., the model assumes random mating, but *A. thaliana* is self-fertilizing). Second, there is always the possibility that results are driven by sampling effects, including demographic history, although using two data sets and separate partitions of those data sets somewhat discounts that notion here. Third, and perhaps most importantly, it is difficult to disentangle genetic from epigenetic effects. Overall, however, this work suggests that gbM has a measurable effect on fitness. The estimated selection coefficients were small (4*N_e_s* = 1.4) but nonetheless similar to the magnitude of selection acting on codon usage that has been measured in *A. thaliana*, *A. lyrata,* and *Capsella rubella* (Qiu et al. 2011).

One interesting feature of codon bias, a phenomenon widely accepted as a genomic feature that is under weak selection, is that it varies among species, with selection detectable in species with large historical population sizes but not detectable in small *N_e_* species (Galtier et al. 2018). An overarching feature of gbM is that much of the experimental and comparative work on gbM has focused on *A. thaliana*. It is worth noting that this species may be atypical in at least three respects. First, two independent studies relying on different data sets and approaches have inferred that *A. thaliana* has lost gbM three times faster than gaining it (Takuno et al. 2017; Muyle et al. 2021) relative to closely related outcrossing species. Second, the recent shift of *A. thaliana* to an inbreeding mating system reduced its effective population size (Mattila et al. 2020), which is likely to have weakened the efficacy of selection on gbM in that species. Finally, methylation mutants usually have little phenotypic effect in *A. thaliana,* whereas they are often lethal in taxa with higher TE load, such as maize (Li et al. 2014). Together these observations suggest that *A. thaliana* may not be the best model for measuring the evolutionary effects of methylation, and yet there is still some evidence that selection acts on gbM in that species, raising the possibility that the effects of gbM may be more pronounced in other species. For this reason, we advocate that similar analyses are extended to other taxa when large methylation data sets become available.

## Does gbM Affect Gene Expression?

**Fig. 2.**
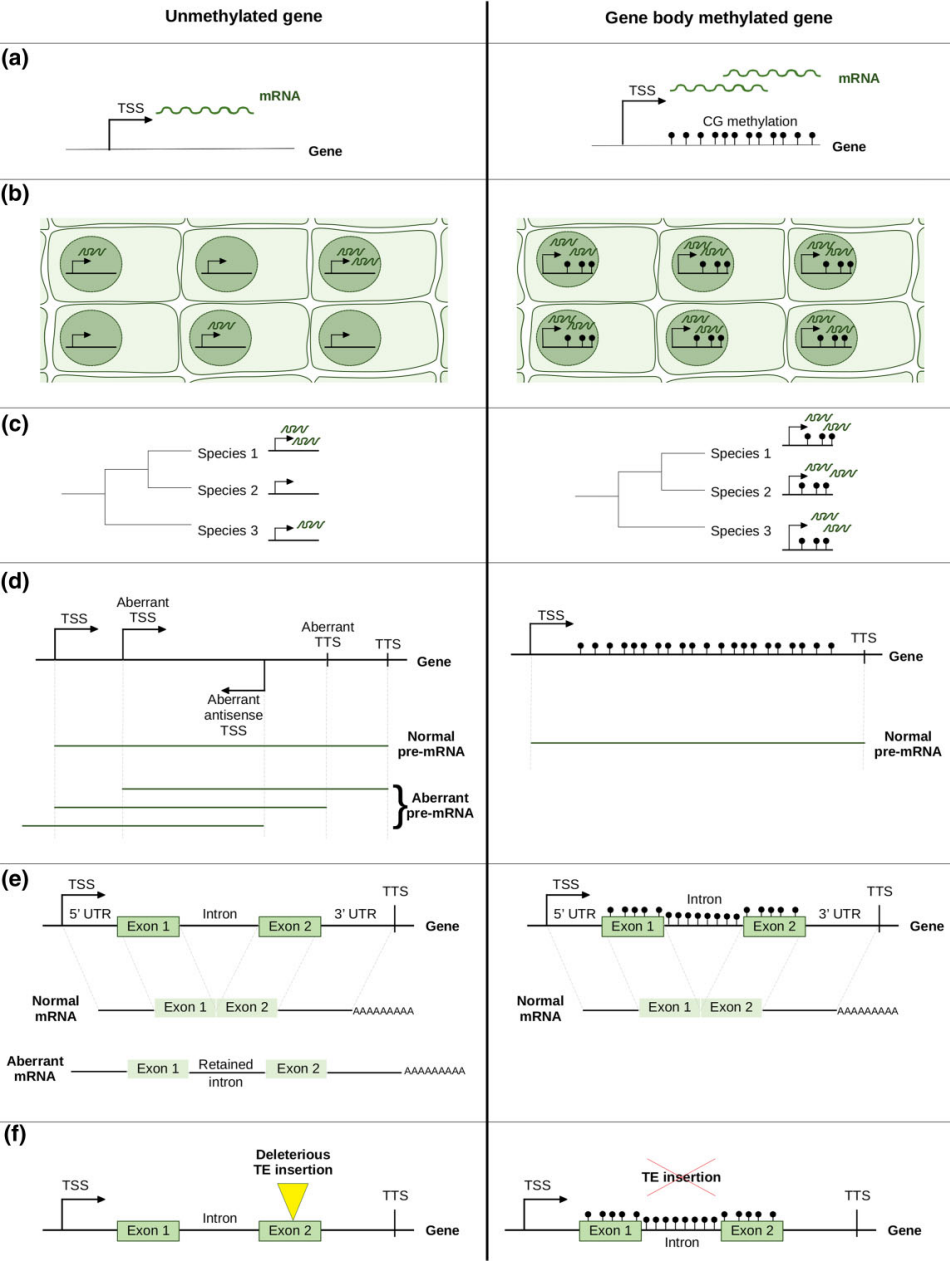
Potential gbM functions and evolutionary consequences. Unmethylated genes, represented on the left column, are compared to gbM genes on the right. TSS stands for the transcription start site, TTS for the transcription termination site, and TE for the transposable element. (*a*) gbM is hypothesized to upregulate gene expression. The number of mRNA molecules, represented by wavy lines, illustrates the gene expression level. (*b*) gbM may stabilize gene expression by triggering consistent expression levels among the cells of a tissue. (*c*) gbM may stabilize gene expression, as seen by the more constant and conserved expression levels observed among species. (*d*) gbM could prevent aberrant internal and reverse transcription by silencing alternative promoters within genes. gbM might also inhibit aberrant TTS. These hypotheses are coherent with the typical depletion of CG methylation observed around the TSS and TTS of genes. (*e*) gbM is hypothesized to facilitate correct splicing and prevent aberrant intron retention. (*f*) Some TEs preferentially insert into genes; however, gbM may protect against deleterious insertions within genes.

Given that there are some indications that gbM may be under weak selection, one naturally wonders what its function might be. One consistent hypothesis has been that gbM affects gene expression levels. This hypothesis first came from the observation that genic methylation levels across genes within *A. thaliana* are associated with expression levels: methylated genes tend to be intermediately to highly expressed, with lower expression variance among tissues (Zhang et al. 2006; Zilberman et al. 2007; Takuno and Gaut 2012). These patterns have been interpreted in two ways: either gbM might affect expression patterns (fig. 2*a*) or, conversely, active transcription might drive gbM (Teixeira and Colot 2009). Many highly expressed genes do not have gbM in *A. thaliana* (Zhang et al. 2006; Zilberman et al. 2007), an observation that discounts the second hypothesis or at least suggests that the relationship is not completely straightforward. Moreover, it is now known that CMT3 does not depend on gene expression to methylate genes but instead on inaccessible chromatin marks and heterochromatin histone variants (Wendte et al. 2019; Papareddy et al. 2021), although it remains possible that the initial recruitment of CMT3 requires or depends on gene expression.

One difficulty in assessing the effect of gbM on gene expression comes from the possible confusion between genetic and epigenetic effects. Indeed, variation in gene expression can be caused by numerous factors—for example, by nearby single nucleotide polymorphisms (SNPs) in regulatory sequences, by a nearby TE insertion (genetic *cis* effects), by a change in a transcription factor (*trans* effects), or by a change in the gene DNA methylation level (epigenetic *cis* effects). Genome-wide association studies (GWASs) and epigenome-wide association studies have shown that DNA methylation variants associated with expression variation are often in linkage disequilibrium with nearby SNPs (Kawakatsu et al. 2016), making it difficult to disentangle the respective contribution of SNPs and methylation variation on gene expression. However, Meng et al. (2016) found a significant association between *cis*-methylation and gene expression in hundreds of genes across 135 *A. thaliana* accessions. Interestingly, gbM was positively correlated with gene expression, and the effect remained significant after controlling for SNPs. Overall, the number and magnitude of the affected loci by DNA methylation were smaller than the effect of SNPs, and hence, the authors concluded that DNA methylation has limited effects on expression variation (table 1; Meng et al. 2016).

The association between gbM and expression was further tested experimentally in epigenetic recombinant inbred lines (epiRILs) obtained through the cross of a *met1* mutant and wild-type (WT) *A. thaliana*, followed by eight generations of inbreeding (Reinders et al. 2009). The resulting epiRILs have a mosaic methylome, with some regions derived from the *met1* mutant that originally lacked gbM and other regions containing CG methylation derived from the WT parent. Bewick et al. (2016) inferred differentially expressed genes between the *met1*-derived regions of epiRILs and their WT homologs. They found only 6 out of 3,471 genes that were gbM in WT plants and became differentially expressed when located in *met1*-derived regions in epiRILs. On the other hand, they found significantly more genes (46 out of 3,124, *P*-value = 2.55 × 10^−9^) that were unmethylated in WT plants and became differentially expressed when located in *met1*-derived regions in epiRILs. Taken together, these results suggest that gbM loss has little, if any, effect on gene expression (table 1). However, if gbM has a small effect on expression, it is likely to have been missed by differential expression analyses that typically detect individual genes with twofold or more expression differences. More subtle effects may be statistically detectable only by approaches that summarize trends across multiple genes. More importantly, the *met1* mutant might be a poor system to study the association between gene methylation and expression level because both methylated and unmethylated genes were upregulated in *met1* mutants using microarray data (Zilberman et al. 2007).

Another approach to test for associations between gbM and expression has been to use comparative and evolutionary rather than experimental approaches. For example, several studies have compared expression between gbM-deprived *E. salsugineum* with its close relative *A. thaliana*, but the results have been controversial. In the first study, Bewick et al. (2016) estimated the expression of unmethylated *E. salsugineum* genes that are orthologous to gbM genes in *A. thaliana* and found no difference in expression between species (table 1). Muyle and Gaut (2019) reanalyzed the data from Bewick et al. (2016) and used genes that were unmethylated in both *A. thaliana* and *E. salsugineum* as a negative control to measure the average difference in expression between the two species. When taking into account this species effect in a linear model, gbM loss in *E. salsugineum* was associated with a small but significant decrease in expression (table 1). In the third study using the same data, Bewick et al. (2019) disagreed on the use of unmethylated genes as a negative control because they have been shown to have more variable expression levels over evolutionary time and again found no effect of gbM loss on gene expression.

Another effort compared gbM and unmethylated genes between *A. thaliana* and *A. lyrata* (Takuno et al. 2017). Methylated genes were expressed at significantly higher levels, on average, and with less variation between species than non-CG-methylated genes. The authors identified genes that changed methylation status between *A. thaliana* and *Arabidopsis lyrata* to examine whether the shift in methylation correlated with gene expression. They found that genes that had gained gbM in one of the two species also tended to shift toward higher expression levels, but these results were not statistically significant (table 1). However, genes that differed in gbM status between *A. thaliana* and *A. lyrata* exhibited significantly higher variance in expression between species than genes that were gbM in both species (Takuno et al. 2017), consistent with previous studies suggesting gbM modulates expression variability (Zilberman 2017). Another comparative study compared the methylomes of eight grass species and found that genes that were gbM in all eight species tended to have higher and less variable expression compared to genes that varied in their methylation state across species (Seymour and Gaut 2020). Although the effect was very small, the results suggested a positive effect of gbM on expression level and expression stabilization (fig. 2*c*). It is worth emphasizing, however, that this approach, like most comparative approaches, cannot determine causality. More recently, we used the 876 *A. thaliana* methylomes to study the association between gbM and gene expression within a species by comparing the methylation state of alleles both to their expression level and to the variability in expression across the larger data subset of 876 *A. thaliana* methylomes (Muyle et al. 2021). Across genes with polymorphic methylation states, the expression of gbM alleles was consistently and significantly higher than unmethylated alleles (table 1). Taken across the entire genome, gbM alleles also had a significantly less variable expression level compared to unmethylated alleles of the same gene (Muyle et al. 2021). Although consistent across the thousands of genes in the data set, the effect was quite small: on average, a methylated allele had ∼1 more RNA-seq read than an unmethylated allele. A weakness of this work is that it did not disentangle potential genetic effects from epigenetic effects; however, the gbM effect did remain consistent when models included a proxy for genetic variation, by including the number of CG dinucleotides in statistical analyses. Consistent with our *A. thaliana* results, work on the outcrossing crucifer *Capsella grandiflora* has revealed that the presence of gbM is a major predictor of *cis*-regulatory constraint (Steige et al. 2017). GbM lowers the probability of allele-specific expression via *cis*-regulation, again suggesting a stabilizing effect of gbM on expression level.

As we have just reviewed, several studies have established an association between gbM and stable expression level (fig. 2*b* and *c*), which complement the proposal that gbM has a homeostatic effect on expression (Zilberman 2017). This phenomenon has been further investigated by Horvath et al. (2019), who studied gene expression levels in *A. thaliana* roots via single-cell RNA-seq. They found no significant correlation between gbM and gene expression noise (as measured by the variation in the expression level among single cells). However, gbM was significantly positively correlated with gene expression consistency, which they measured as the number of single-cell RNA-seq replicates in which the gene was expressed (fig. 2*b*). This effect remained after correcting other genomic features such as gene expression, gene length, gene conservation, and gene duplication status. Therefore, Horvath et al. (2019) found that gbM genes are more consistently expressed than unmethylated genes across cells of a tissue, which can be interpreted as implying that gbM is involved in the maintenance of a consistent gene expression (fig. 2*b*). If this is true, the mechanism by which this happens remains unknown. One hypothesis comes from the anticorrelation observed between genome-wide distributions of the histone variant H2A.Z and DNA methylation in *A. thaliana* (Zilberman et al. 2008). H2A.Z is typically associated with transiently expressed response genes, such as immune-responsive or environmental stimulus-responsive genes (Coleman-Derr and Zilberman 2012). In *met1* mutants, the loss of DNA methylation was accompanied by a gain in H2A.Z deposition (Zilberman et al. 2008). However, in an *h2a.z* mutant, DNA methylation patterns were only minimally affected (Coleman-Derr and Zilberman 2012), suggesting that DNA methylation prevents H2A.Z incorporation but not the converse. Based on these observations, it has been proposed that gbM serves to stabilize transcription by preventing deposition of the histone variant H2A.Z (Coleman-Derr and Zilberman 2012).

Finally, Shahzad et al. (2021) have used quantitative trait loci (QTL) mapping to identify ∼1,000 genes for which the proportion of methylated CG sites significantly correlates with expression level across a sample of over 900 natural *A. thaliana* accessions. The variance in expression explained by CG methylation is modest for most genes, but for some genes, it reaches levels comparable to the effect of SNPs on expression. gbM is mostly positively correlated with expression; in contrast, TE-like methylation (i.e., in all three contexts) is, as expected, negatively correlated with expression. In a clever extension to control the effect of linked SNPs, Shahzad et al. (2021) identified SNPs with significant effects on expression using GWAS. They then repeated the analysis linking CG methylation and expression within nested sets of accessions that carry the same GWAS allele. For the vast majority of genes, this approach confirmed the significant positive correlation between gbM and expression level, either because there was no GWAS SNP or because at least one nested sample had a significant correlation. A second control analyzing haplogroups, which corrects for *cis* genetic variation, led separately to the same conclusion. They then studied gene expression in the *met1 A. thaliana* mutant without gbM, and they found as expected a reduced expression level in genes with these three characteristics: (1) genes with a significant positive correlation between CG methylation and expression level, (2) genes that were methylated in the WT Col-0 accession (which is the accession used for the *met1* mutant), and (3) genes for which the effect of gbM was not confounded by linked genetic variants. The authors concluded that gbM is positively correlated with gene expression in hundreds of genes, independently of local genetic variants.

Although Shahzad et al. (2021) did attempt to account for *trans* effects as well as *cis* effects, a shortcoming of most of the comparative studies referred to in this section is that they do not account for possible *trans* genetic effects on gene expression, which may result in overestimated *cis* epigenetic effects. Altogether, however, evolutionary and comparative studies tend to find small but detectable relationships between gbM and either gene expression levels or gene expression variation. These results contrast with many direct experimental measurements of gbM based on *A. thaliana* mutants. If the comparative conclusions are correct, they are important because they suggest that gbM has a phenotype that may be the target of natural selection.

## Potential Effects of gbM on Internal and Reverse Transcription

To date, studies have been inconsistent as to whether gbM associates with gene expression (table 1). When it does associate with expression, it is also difficult to disentangle cause from effect. If, however, we assume there is a real relationship between gbM and gene expression, there remains an open question: what is the mechanism(s) by which gbM affects expression? One hypothesis is that gbM improves transcription by regulating alternative promoters within gene bodies, thereby potentially preventing aberrant internal and/or antisense transcription (fig. 2*d*; Tran et al. 2005; Maunakea et al. 2010). This hypothesis stems from the observation that CG methylation is typically depleted within active promoter regions (Feng et al. 2010) and also that genes with CG-methylated promoters are silenced (Niederhuth et al. 2016). Aberrant transcription, whether in the sense or antisense orientation, is expected to be deleterious because it is energetically costly and leads to the accumulation of both unnecessary transcripts and truncated proteins that can be toxic for the cell. Aberrant antisense transcription is expected to disturb gene expression because RNA polymerases coming from both directions may collide. Moreover, the RNA-directed DNA methylation pathway can be activated by the pairing of sense and antisense transcripts into double-stranded RNA (Tran et al. 2005), which may further prevent gene expression. Hence, if gbM prevents aberrant reverse transcription, it could explain the aforementioned association between gbM and gene expression.

However, the results of tests for this effect have been inconsistent (table 1). Some of these tests have taken place in mammalian systems because they too exhibit CG methylation within genes (Yi 2017), even though they mostly do not methylate in the CHG and CHH contexts. For example, Neri et al. (2017) studied mouse embryonic stem cells in *DNA methyltransferase 3b* (*Dnmt3b)* mutants that lack gbM and compared them to WT. To quantify internal transcription, the authors used a ratio of the number of RNA-seq reads that map to the third exon divided by the number of reads mapping to the first exon (hereafter exon3/exon1). This ratio is expected to increase when there is cryptic intragenic initiation of transcription. Neri et al. (2017) found that the loss of gbM was accompanied by higher exon3/exon1 ratios than WT mice, suggesting an increase of spurious internal transcription between exons 1 and 3 when gbM is lost (table 1). The results were confirmed by a sequencing technique that allows characterization of the exact position of the 5′ end of mRNAs by targeting the mRNA cap (DECAP-seq). However, Teissandier and Bourc’his (2017) performed similar analyses in *Dnmt* triple mutants on highly expressed genes, and they were unable to corroborate the findings of Neri et al. (2017) (table 1). Results from Teissandier and Bourc’his (2017) suggest that the role of gbM in suppressing spurious transcription initiation may be specific to the lack of DNMT3B, but only while other DNMTs are still present.

Similar work has sought evidence for an effect of gbM on aberrant transcription in plants. For example, Bewick et al. (2016) compared *met1*-derived regions of *A. thaliana* epiRILs with orthologous WT regions. They quantified antisense transcription and found that gbM loss did not lead to an increase in differentially expressed antisense transcripts (table 1). However, Choi et al. (2020) detected that the expression of antisense transcripts was activated in 938 genes in *h1*, *met1* double mutants compared to WT. The number of upregulated antisense transcripts was comparatively low in single mutants when compared with WT (145 and 34 for *met1* and *h1*, respectively), suggesting redundancy in H1 and MET1 repression of antisense transcription. This finding demonstrates that, at least for some genes, gbM may repress antisense transcription in *A. thaliana* jointly with histone H1 (table 1). This study also again exemplifies redundancy among DNA methylation, histone variants, and histone marks. These different epigenetic marks are interdependent and play overlapping roles in the cell, complicating the characterization and inference of potential gbM effects.

More recently, Li et al. (2021) used RNA long reads sequenced by Oxford Nanopore Technology Direct Sequencing (ONT DRS) to characterize transcription start sites (TSSs) in *A. thaliana*. They found that the *met1–3* mutant, which lacks CG methylation, has significantly more unique TSSs compared to WT, and these unique TSSs occurred in regions where mutant methylation was lower than WT. These results suggest that gbM can prevent the initiation of aberrant transcription. The transcription termination site (TTS) was also affected by DNA methylation (Li et al. 2021). Indeed, the *met1–3* mutant had a higher number of unique TTS than WT, indicating that CG methylation also inhibits aberrant transcription termination. Altogether, this work suggests that gbM could ensure proper transcription of genes from start to end (fig. 2*d*).

Here, we revisited this issue by analyzing Isoseq (PacBio RNA long read) data in maize and *A. thaliana* (Supplementary Materials and Methods, Supplementary Material online). We included maize because this is (to our knowledge) the first such attempt to examine this question in a plant other than *A. thaliana.* We focus on Isoseq data because it can represent full-length mRNA, thanks to the selection of mRNAs that contain a 3′ poly-A tail and, in some cases, a 5′ cap (when sequencing is done with a cap-trap step). The *A. thaliana* Isoseq data set we analyzed has a 5′ cap so that most Isoseq reads likely represent full-length mRNAs (Supplementary Materials and Methods). Some of the *A. thaliana* data set was publicly available (Cartolano et al. 2016), and we also generated new Isoseq data for this study; in both cases, the data were generated from Col-0 inflorescences. In contrast, the maize Isoseq data, which was generated on pooled RNA extracted from six tissues at different developmental stages of the B73 inbred line (Wang et al. 2016), were not generated with a cap-trap step. With both the maize and *A. thaliana* data sets, we considered aberrant internal transcription to be reflected in Isoseq reads that begin after the start of exon 1 (fig. 2*d*). For each gene, the proportion of full-length Isoseq reads with a “conventional” TSS (i.e., that begin prior to the start of exon 1) was computed and compared between gbM and unmethylated (UM) genes. gbM and UM genes were categorized from publicly available methylation data (see Supplemental Materials and Methods, Supplementary Material online).

**Fig. 3.**
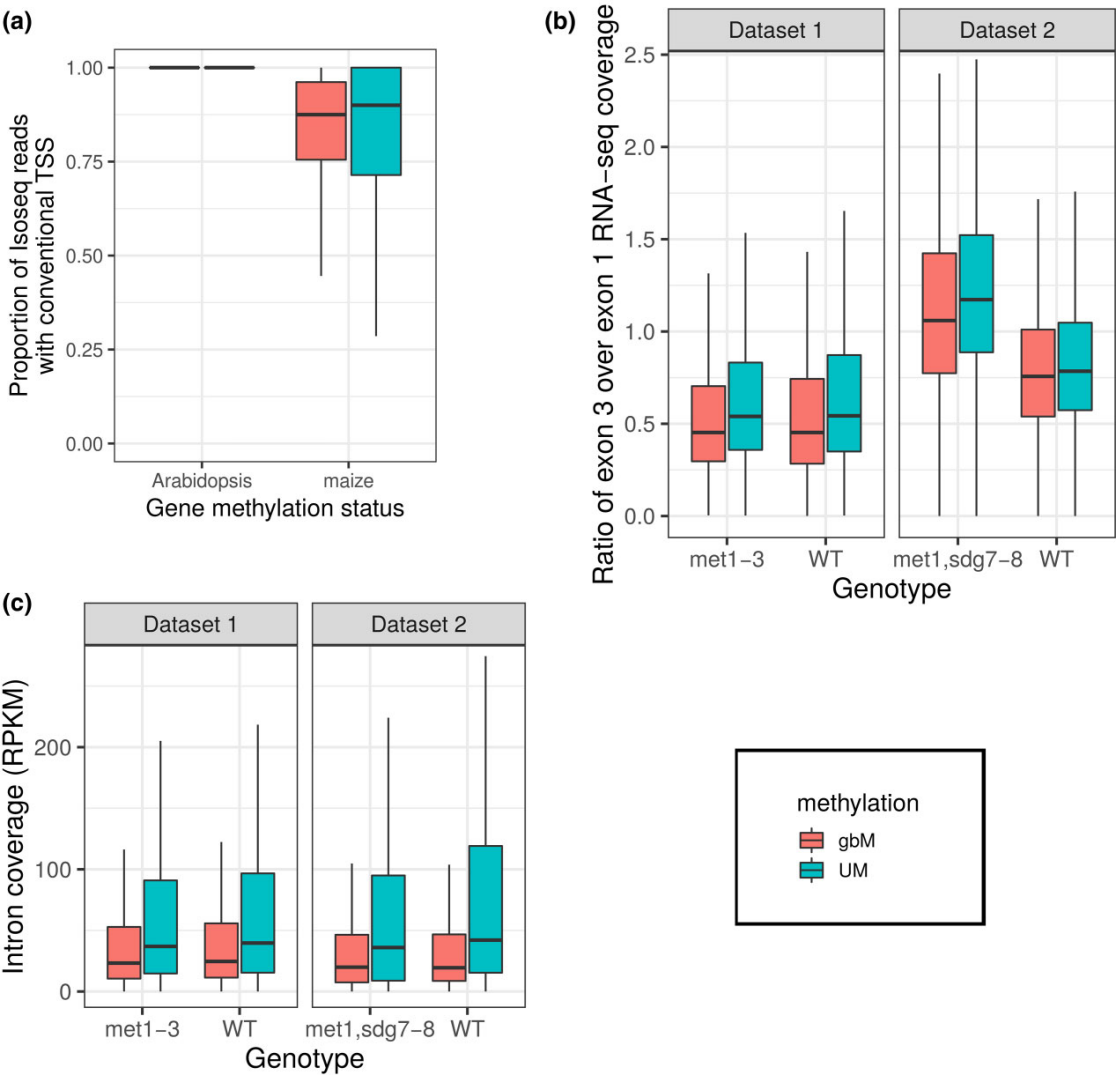
Novel analyses to assess the effect of gbM on transcripts. (*a*) Proportion of full-length Isoseq reads with conventional TSS in gbM and UM genes in maize and *A. thaliana*. Isoseq reads that started after the start of exon 1 were considered as nonconventional. (*b*) RNA-seq read coverage ratio between exon 3 and exon 1 for gbM and UM genes in *A. thaliana*. Internal transcription starts happening between exon 1 and exon 3 and is expected to increase the ratio of exon 3 to exon 1 coverage. (*c*) RNA-seq read coverage of introns (in RPKM) for gbM and UM genes in *A. thaliana*. Pools of gbM genes are drawn in red, and those of UM genes are drawn in turquoise. In data set 1, WT controls were compared to *met1-3* mutants. In data set 2, other WT controls were compared to *met1,sdg7–8* triple mutants. The boxplots show the median, the hinges are the first and third quartiles (the 25th and 75th percentiles), and the whiskers extend from the hinge to the largest or smallest value no further than 1.5 times the interquartile range (distance between the first and third quartiles).

In maize, we found that gbM genes had a significantly higher proportion of conventional TSSs (average 0.81) compared to UM genes (average 0.78, Wilcoxon test *P*-value = 5.63 × 10^−11^fig. 3*a*); superficially, this observation complies with the prediction that gbM genes have less aberrant transcription. However, gbM is known to be associated with more highly expressed and longer genes (Zhang et al. 2006; Takuno and Gaut 2012), and these covariates must be taken into account. Genes with higher expression had a significantly higher proportion of conventional TSSs (generalized linear model contrast estimate = 0.042, *Z*-ratio = 69.15, *P*-value <2 × 10^−16^, Supplementary table S1, Supplementary Material online), perhaps reflecting higher selective pressures to remove aberrant transcription for highly expressed genes. Longer genes had significantly fewer conventional TSSs (generalized linear model contrast estimate = −0.178, Z-ratio = −130.7, *P*-value <2 × 10^−16^Supplementary table S1, Supplementary Material online), which could be attributable to the higher probability of a long gene harboring an aberrant internal promoter or an experimental artifact (i.e., longer genes may have a higher chance of having their mRNA not fully reversed transcribed during sequencing, leading to 5′ truncated transcripts and wrongly inferred aberrant TSS). Notably, however, only 321 of 2059 detected nonconventional TSSs occurred in introns. After taking gene length and gene expression into account in a generalized linear model (Supplementary Materials and Methods, eq. 6, Supplementary Material online), UM genes had a significantly higher proportion of conventional TSSs compared to gbM genes (generalized linear model contrast estimate = 0.214, *Z*-ratio = 33.4, *P*-value <2 × 10^−16^, Supplementary table S1, Supplementary Material online). This result is not consistent with the expectation that gbM prevents aberrant TSS (table 1).

The *A. thaliana* results complement the maize results in some ways but not others. Since the data were generated with a 5′ cap, the overall proportion of conventional TSSs in the *A. thaliana* Isoseq data set was higher than in maize (fig. 3*a*). gbM genes had a lower proportion of conventional TSSs (mean 0.90) compared to UM genes (0.96). However, after taking gene length and gene expression into account in a generalized linear model, gbM genes did have significantly more conventional TSS compared to UM genes (*P*-value <2 × 10^−16^, Supplementary table S2, Supplementary Material online). We conclude that the Isoseq data do reflect some advantage of gbM in terms of avoiding internal transcription start in WT *A. thaliana* but not in maize (table 1).

We explored these ideas further by turning to a different approach that relies on RNA-seq reads in gbM mutants. Because 5′ cap Isoseq data are not available for methylation mutants, we inferred internal transcription starts using the RNA-seq coverage ratio of exon3/exon1, following the approach of Neri et al. (2017) (see Supplementary Materials and Methods, Supplementary Material online). If gbM prevents aberrant internal transcription start, this ratio should increase in gbM mutants relative to WT. We therefore measured exon3/exon1 RNA-seq coverage in WT and gbM mutants. We performed this comparison for two data sets based on two different gbM mutants. In data set 1, RNA-seq data were generated on 13-day-old seedlings for three replicates of WT controls that were compared to three replicates of *met1–3* mutants (Zhang et al. 2017). In data set 2, RNA-seq data were generated on leaf tissue for three replicates of WT controls (these differed from the controls within data set 1) that were then compared to five replicates of *met1,sdg7–8* triple mutants (Bewick et al. 2016). In WT plants, gbM genes had a lower exon3/exon1 coverage ratio compared to UM genes (fig. 3*b*), suggesting superficially that gbM could prevent internal transcription start. This was observed consistently for WT plants from data set 1 (mean exon3/exon1 coverage 0.662 in gbM genes, 1.919 in UM genes, one-sided Wilcoxon test *P*-value <2.2 × 10^−16^) and also from data set 2 (mean exon3/exon1 coverage 1.013 in gbM genes, 3.684 in UM genes, one-sided Wilcoxon test *P*-value = 1.3 × 10^−11^). However, this same difference between gbM genes (as defined in WT plants) and UM genes was also apparent in mutant plants that lacked gbM (fig. 3*b*). That is, gbM genes had a lower exon3/exon1 ratio compared to UM genes in *met1–3* mutants (0.512 vs. 1.876, one-sided Wilcoxon test *P*-value <2.2 × 10^−16^) and in *met1,*s*dg7–8* triple mutants (1.232 vs. 1.566, one-sided Wilcoxon text *P*-value <2.2 × 10^−16^). These observations suggest that the fact that gbM genes have lower exon3/exon1 coverage in RNA-seq data is not due to their methylation state alone because the same pattern is observed in mutants without gbM. While one must always be careful that mutants can be complex and may reflect other (unknown) effects, the data again provide little support for the notion that gbM alone prevents aberrant internal transcription initiation in *A. thaliana* genes (table 1). Interestingly, Choi et al. (2020) showed that gbM and H1 play a redundant role in inhibiting aberrant reverse transcription, and Martin et al. (2021) hypothesized that another epigenetic mark, CHH islands, may have some redundant function with gbM. These redundancies could explain why we did not detect any change in internal transcription start in *A. thaliana* gbM mutants because these redundant epigenetic marks may have been functioning in gbM mutants, thus complicating inferences about gbM effects.

Another aspect of aberrant transcription, aside from internal transcription initiation, is reverse transcription. We again used the Isoseq data from *A. thaliana* to estimate the proportion of full-length antisense reads, which correspond to reverse transcription events (see Supplementary Materials and Methods, Supplementary Material online). gbM genes had a significantly lower mean proportion of antisense reads (average 0.0046) compared to UM genes (average 0.016, Wilcoxon test *P*-value = 3.16 × 10^−12^). This result held after accounting for gene length and gene expression in a generalized linear model (Supplementary table S3, Supplementary Material online). However, in maize Isoseq data, the gbM genes had significantly more antisense transcription compared to UM genes (Supplementary table S4, Supplementary Material online). We conclude that there is evidence that gbM prevents antisense transcription in *A. thaliana* based on Isoseq data from WT plants (table 1), but we have uncovered no such evidence in maize. Altogether, however, we believe there are enough compelling observations—both from animals and from *A. thaliana* plants (Choi et al. 2020)—to suggest that further dissection of this potential function may be worthwhile.

## Assessing the Effect of gbM on Splicing Fidelity

Another hypothesis is that gbM improves splicing fidelity and prevents aberrant intron retention (fig. 2*e*), but this raises the question of how splicing fidelity may drive a relationship between gbM and gene expression. One possibility is that poor splicing in the absence of gbM leads to the retention of introns (fig. 2*e*) that contain premature stop codons. Aberrant transcripts containing premature stop codons are typically sent to the nonsense-mediated mRNA decay for destruction (Causier et al. 2017), which might in turn lower gene expression. This suggests a potential relationship among gbM, splicing fidelity, and gene expression.

The effect of gbM on splicing fidelity has been tested across various taxa, and the results have been—like studies of aberrant transcription—somewhat inconsistent. In honeybee and mouse embryonic stem cells, for example, it is clear that alteration of DNA methylation impacts alternative splicing (Lev Maor et al. 2015). In honeybees, DNA methylation is predominantly on gene bodies in the CG context. A knockdown of the expression of *dnmt3*, which is required for de novo DNA methylation, decreased global genomic methylation level and caused widespread changes in alternative splicing in fat tissue (Li-Byarlay et al. 2013). In mouse embryonic stem cells, Yearim et al. (2015) constructed an experimental system in which differential DNA methylation could be limited to a single gene while all other cellular factors remained identical. Using this system, they demonstrated a direct causal relationship between DNA methylation and the recruitment of splicing factors. Patterns of methylation near splice sites have also been studied in maize, where CHG methylation of the splicing acceptor site is associated with a lower efficiency of splicing and CHH methylation does not correlate with splicing efficacy (Regulski et al. 2013). Surprisingly, however, the effect of CG methylation was not tested explicitly. Horvath et al. (2019) followed the maize work by measuring splicing fidelity in *A. thaliana*. They found that gbM was negatively correlated with the amount of RNA-seq reads that map to introns, suggesting that gbM genes tend to retain fewer introns in their mRNA compared to UM genes. Similarly, Li et al. (2021) recently used ONT DRS to characterize splicing in *A. thaliana*. They found that retained introns had significantly lower CG methylation levels around their splicing sites (both donor and acceptor sites) compared to spliced introns in WT and some CHG and CHH methylation mutants. This suggests that gbM facilitates splicing. However, Bewick et al. (2016) found no evidence for this splicing effect when they compared *met1* epiRILs to WT plants. In fact, they found that WT gbM genes retained significantly fewer intron reads than UM genes after they lost gbM in the *met1* background. This work suggests that this intron effect is a property of the genes rather than gbM per se.

Given contradictory results in the literature, we further tested the hypothesis that gbM prevents aberrant intron retention using *A. thaliana* Isoseq data (Supplementary Material and Methods, Supplementary Material online). The proportion of full-length Isoseq reads that retained at least one intron was higher in gbM genes (mean 0.149) compared to UM genes (mean 0.106). This result remained significant after taking gene length and gene expression into account in a generalized linear model (table 1, Supplementary table S5, Supplementary Material online). We also measured intron RNA-seq coverage in WT and mutant *A. thaliana* plants that lack gbM (Supplementary Material and Methods, Supplementary Material online). Similar to Horvath et al. (2019), we found that gbM genes had a lower intron read coverage compared to UM genes in WT plants (fig. 3*c*) both in data set 1 (mean gbM genes intron coverage 61.51 RPKM, vs. 133.42 for UM genes, one-sided Wilcoxon test *P*-value < 2.2 × 10^−16^) and in data set 2 (mean gbM genes intron coverage 53.13 RPKM, vs. 208.68 in UM genes, one-sided Wilcoxon test *P*-value < 2.2 × 10^−16^). However, the difference between gbM genes and UM genes was also found in mutant plants that lack gbM (fig. 3*c*). Indeed, gbM genes had a lower intron read coverage compared to UM genes in met1–3 mutants (59.04 vs. 131.65 RPKM, one-sided Wilcoxon test *P*-value < 2.2 × 10^−16^) and in met1, sdg7–8 triple mutants (51.27 vs. 132.97 RPKM, one-sided Wilcoxon test *P*-value < 2.2 × 10^−16^). This again suggests that the fact that gbM genes have lower intron coverage in RNA-seq data is not due to their methylation state alone (table 1). Another possibility is again that some other epigenetic mark plays a redundant role with gbM in preventing aberrant intron retention. In summary, there is not yet a clear consensus, or even a clear trend, as to whether gbM plays a role in splicing fidelity. However, we note again that most of the work in plants has focused on *A. thaliana*, and, as previously discussed, it may be helpful to extend these analyses to other species. The question of the potential role of gbM in splicing fidelity, like its role in aberrant transcription, remains unresolved and will benefit from the broader and more comprehensive investigation.

## Potential Relationship between gbM and TE Insertion

Another hypothesized function of gbM is that methylation protects against the insertion of some TEs (fig. 2*f*). This hypothesis primarily stems from two studies in maize that focused on Robertson’s Mutator (Mu) transposons, which typically insert within or near genes and can be highly deleterious by disrupting gene function. In the first study, Liu et al. (2009) found that Mu transposons insert preferentially within unmethylated regions of the B73 genome. However, the methylation context could not be determined, which motivated Regulski et al. (2013) to repeat the analyses with context-specific DNA methylation data. They found that Mu transposon insertion sites within genes were strongly depleted in CG-methylated regions (table 1), but these regions were not depleted in CHG nor CHH methylation relative to average gene methylation. This raises the possibility that gbM is beneficial because it deters transposon insertions. This hypothesis is also difficult to disentangle from covariates, particularly the observation that gbM genes tend to be under stronger selective constraint than unmethylated genes, as measured by nonsynonymous divergence (Takuno and Gaut 2012), although there is conflicting evidence that gbM genes do (Niederhuth et al. 2016) or do not (Takuno and Gaut 2013) evolve more rapidly in the Poaceae. Nonetheless, it is possible that the apparent difference in TE insertion reflects different strengths of selection on gbM versus unmethylated genes, rather than a direct effect of gbM on TE insertion rate.

It would be insightful to repeat analyses of the effect of DNA methylation on TE insertion in other plant species and with different TE types to test for the potential broader relevance of this idea. If this phenomenon occurs across diverse species and TE types, it could partially explain the link between gbM and expression stabilization within and between species. Indeed, a genic TE insertion might prevent proper gene expression because silencing of the TE by methylation in the three contexts might spread to the gene and affect expression (Choi and Lee 2020). Also, if the TE is inserted within an exon, it might lead to the appearance of premature stop codons and the destruction of mRNA by nonsense-mediated mRNA decay. This would lead to more expression variation among accessions of a species and also among species (fig. 2*c*).

## Conclusions and Future Research Directions

The molecular mechanisms leading to gbM establishment and maintenance in plants have been remarkably well elucidated (fig. 1). However, the function and potential importance of gbM remain debated, as illustrated in this review by the numerous studies that are inconsistent or, in some cases, contradictory (table 1). The main sources for this persistent uncertainty come first from the difficulty in disentangling epigenetic from genetic effects and second from the complex system of redundancies and overlapping functions among gbM, histone variants, and other epigenetic marks. These dependencies and redundancies undoubtedly complicate the interpretation of experimental mutants, as illustrated by contrasting results based on *met1* plants with or without *h1* mutation (table 1; Bewick et al. 2016; Choi et al. 2020).

Despite these difficulties, there has been substantive progress toward understanding the effect, dynamics, and potential adaptive impact of gbM. In the last few years, several studies have concluded that gbM is associated with both the level and the variance of gene expression (table {PI}1). An interesting corollary of these observations is that many experimental efforts to measure this association based on *A. thaliana* mutants have yielded negative results (table 1). While these experiments may reflect reality, our view is that experimental approaches have nonetheless suffered from a few common shortcomings. First, we suspect (but certainly do not know) that the reliance on *A. thaliana* is limiting; it is illogical to expect a strong experimental effect in a system that is studied in part because methylation mutants are viable and thus may not have a strong effect relative to other plant systems. Second, as noted above, it is not clear that all mutants are equivalent because some mutants may be unsuitable for detecting specific effects due to dependencies and redundancies. Finally, it can be exceedingly difficult to identify subtle effects using short-term experimental approaches. Unfortunately, however, the inability to detect an effect is often incorrectly interpreted as the absence of an effect.

In contrast, evolutionary and comparative approaches based on genetic diversity or species comparison have often, but not always, found an association between gbM and gene expression, particularly expression homeostasis (table 1). These analyses also suffer from a number of potential drawbacks, including reliance on simplified models, discrete definitions of which genes are (or are not) gbM, and an inability to disentangle causation from correlation. One potential reason for detecting an effect using these approaches is that even very subtle effects can accrue over time and thus be detected by evolutionary contrasts. It is difficult to establish whether these associations are causal due to all the complex reasons cited above—that is, functional redundancies among epigenetic marks and difficulties in discriminating genetic from epigenetic effects. Nonetheless, the apparent association between gbM and expression is important, because it provides a potential phenotype on which selection can act. Although more investigation is needed to test whether gbM is shaped by selection, both phylogenetic and population genetic studies suggest that selection acts to maintain gbM status in some genes. Moreover, population variation in gbM has been shown to associate with fitness under water stress and selection for flowering time (Shahzad et al. 2021). These fitness effects appear to stem from a correlation between gbM and gene expression, as demonstrated by the fact that experimental modification of candidate gene expression affects the trait under study (Shahzad et al. 2021). Altogether, these results suggest that gbM may affect fitness and phenotype through an effect on gene expression, thereby potentially affecting species adaptation independently of genetic variation. We emphasize, however, that these effects are subtle, at best, and so, there is still much to learn, even about the simple question as to whether and when gbM associates with gene expression (table 1).

**Fig. 4.**
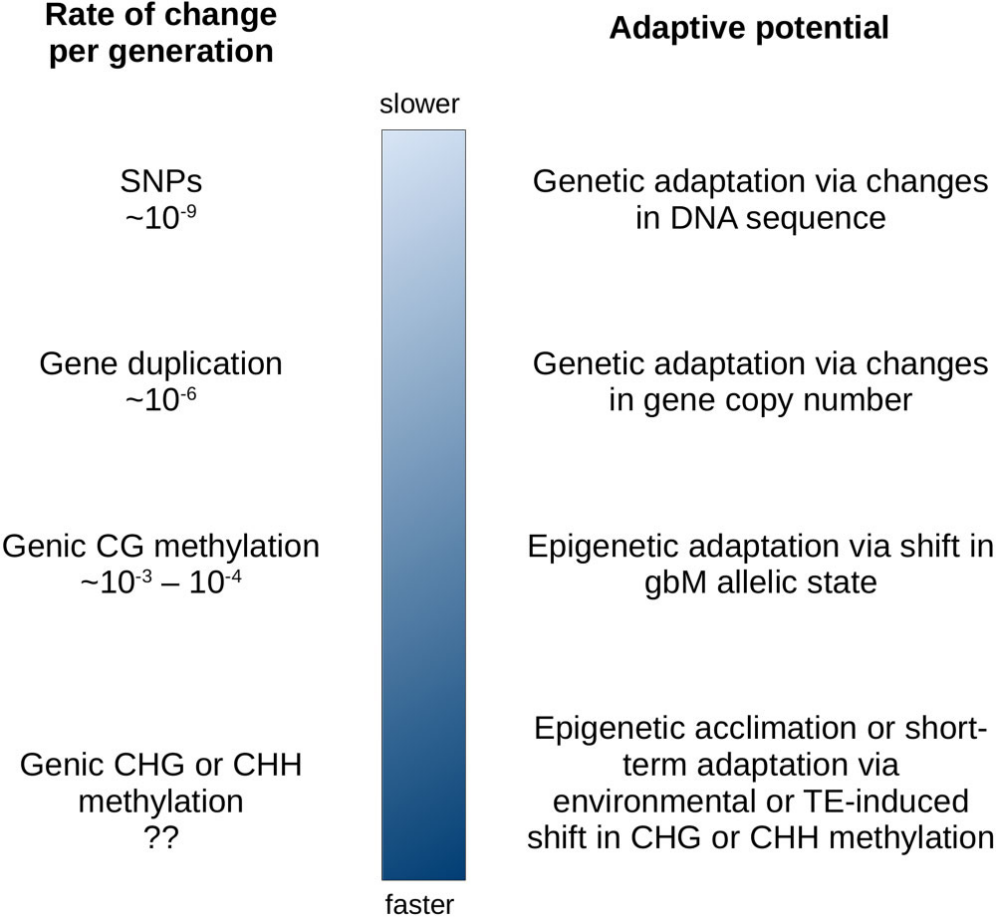
Tempo of epigenetic versus genetic change. The rates of change come from a series of sources (Jelesko et al. 2004; Ossowski et al. 2010; Gaut et al. 2011; van der Graaf et al. 2015).

If there is selection on gbM itself—or on another epigenetic feature that correlates with gbM—then this fact may be the basis for an “important conceptual change in evolutionary biology” (Cavalli and Heard 2019) because selection on epigenetic modifications has the potential to affect the timeframe of mutation and thus, potentially, of adaptative change. As an example, figure 4 illustrates the tempo of epigenetic versus genetic change. As we have noted, epigenetic change can be incredibly rapid. For example, CHH methylation is reset every generation, making it unlikely to be under direct selection because it is not transmitted transgenerationally. CHH methylation (and to some extent CHG methylation) is perhaps better described as tracking genetic (i.e., TE and CMT3) activity. In contrast, CG methylation is heritable and mutates approximately three and six orders of magnitude faster than gene duplication and nucleotides, respectively (fig. 4). It is worth noting, however, that the rate of change of an entire gene or allele from gbM to UM is substantially slower because it probably requires numerous changes of individual sites. The rate of change for an entire allele, based on our previous SFS analysis in *A. thaliana* populations, is ∼3 × 10^−7^ per gene per generation (Muyle et al. 2021), an estimate based on models that require assumptions about the effective population size but, if accurate, is still faster than the rate of nucleotide change. In theory, then, methylation variation may provide a rapid source of phenotypic novelty that could be subjected to natural selection on rapid—and perhaps even ecological—time scales.

## Supplementary Material

evac038_Supplementary_Data

## Data Availability

*Arabidopsis thaliana* Isoseq data newly sequenced here were deposited in NCBI under BioProject accession PRJNA754773.
