# Supplementary material for: Gene Body Methylation in Plants: Mechanisms, Functions, and Important Implications for Understanding Evolutionary Processes
Source: Genome Biol Evol. 2022 Mar 17;14(4):evac038. doi: 10.1093/gbe/evac038 (PMC8995044; doi:10.1093/gbe/evac038)
Supplement: evac038_Supplementary_Data [file evac038_supplementary_data.pdf]

Gene-body methylation in plants: mechanisms, functions and important implications for understanding evolutionary processes

Review

Aline M. Muyle[1], Danelle K. Seymour[2], Yuanda Lv[3], Bruno Huettel[4], Brandon S. Gaut[1]

[1]Ecology and Evolutionary Biology, UC Irvine, Irvine.

[2]Botany & Plant Sciences, UC Riverside, Riverside, United States.

[3]Provincial Key Laboratory of Agrobiology, Institute of Crop Germplasm and Biotechnology, Jiangsu Academy of Agricultural Sciences, Nanjing, China.

[4]Max Planck Genome Centre Cologne, Max Planck Institute for Plant Breeding, Cologne, Germany

Supplementary Materials and Methods

Isoseq sequencing

Total RNA was extracted from inflorescenses of Arabidopsis thaliana (Col-0) and quality assessed by Nanochip (Agilent Bioanalyser, Santa Clara, U.S.A.). Next, cDNA was synthesized according to the TeloPrime Version 2 kit (Lexogen, Vienna, Austria). We exchanged the Lexogen first-strand synthesis oligo-dT primer by the primer (5'-AAGCAGTGGTATCAACGCAGAGTACNNNNNNNNNNT(30)VN-3') to introduce a 3' anchor base and a unique molecular identifier sequence (UMI) by including N(10) with N=A, C, G, or T at even frequency. Then the optimal number of cycles was determined by qPCR with fluorochrome 1x Evagreen (Biotium, Fremont, U.S.A.), the TeloPrime kit chemistry and 25% of the cDNA as input. The residual cDNA was mass amplified with a cycle number where 80% of the maximal fluorescence signal has been reached. PCR products were bead purified (AMPure beads, Beckman-Coutler, Brea, U.S.A.)

followed by PacBio library preparation with the SMRTbell Template Prep Kit 1.0-SPv3 (Pacific Biosciences, Menlo Park, U.S.A.). Long-read sequencing was performed with a Sequel sequencer with a 3.0 Binding kit, 3.0 sequencing chemistry, and a single 1Mv3 SMRT cell (Pacific Biosciences, Menlo Park, U.S.A.). The movie time was 20 hours after a 2 hours immobilization step and 1 hour pre-extension step to adjust for circular consensus sequencing (CCS) mode.

Publicly available datasets

Isoseq data for *A. thaliana* was retrieved from Genbank, accession PRJNA306427 (Cartolano et al. 2016). IsoSeq data in maize, accession number SRP067440, comprised 643,330 full-length high-quality consensus transcript sequences (Wang et al. 2016).

A. thaliana RNA-seq data was retrieved from SRA study number SRP101919 (Zhang et al. 2017), which included three biological replicates of 13 days old met1-3 mutants and three wild-type (WT) controls. RNA-seq data was also retrieved for SRA study number SRP066358 (Bewick et al. 2016), which included five replicates of met1,sdg7-8 triple mutant and three replicates of WT control sampled for leaf tissue.

Maize BS-seq data came from Seymour *et al.* (2019). *A. thaliana* Col-0 BS-seq data came from Bewick *et al.* (2016).

Inference of cytosine methylation

Cytosine methylation calls and gene methylation states were inferred as in Muyle *et al.* (2021).

Analysis of Isoseq data

Each run of the *A. thaliana* Isoseq data from Cartolano *et al.* (2016) was analyzed separately using the Pacific Biosciences' SMRT Tools version 6.0. Circular consensus sequences were generated from subreads using ccs tool (with parameters --minPredictedAccuracy 0.8 --minReadScore 0.65 --

richQVs --polish --minPredictedAccuracy 0.8 --minPasses 0 --maxLength 21000 --minLength 50). The Iso-Seq classify tool was then used to separate the reads into full length and non-full length, non-chimeric, reads (using parameter --min_seq_len 200). Full-length reads were defined as containing both 5’ and 3’ cDNA primers and a poly-A tail. Primers from the Clontech SMARTer kit or primers from the Lexogen TeloPrime kit were used depending on the kit used to generate the cDNA library. The Iso-Seq cluster tool was then used to cluster all full-length reads derived from the same transcript isoform using the default minimum Quiver accuracy setting (0.99). The non-full-length reads were used to polish the consensus sequences produced by the Iso-Seq cluster tool using the Quiver algorithm.

The newly sequenced *A. thaliana* Isoseq runs were analyzed separately using the Pacific Biosciences' SMRT Tools version 7.0.0. Circular consensus sequences were generated from subreads using ccs tool (with parameters --minPredictedAccuracy 0.8 --minReadScore 0.65 --richQVs --polish --minPredictedAccuracy 0.8 --minPasses 0 --maxLength 21000 --minLength 50). The lima and isoseq3 refine tools were then used to isolate full length non-chimeric reads. Full-length reads were defined as containing both 5’ and 3’ cDNA primers and a poly-A tail (option --require-polya). Primer sequences from the Lexogen TeloPrime kit were used. The isoseq3 cluster and polish tools were then used to cluster all full-length reads derived from the same transcript isoform and polish them using subreads. Only high quality transcripts were kept for further analyses. Transcripts of the two *A. thaliana* Isoseq datasets sequenced with cap trap (TeloPrime kit) were pooled before mapping.

The Arabidopsis thaliana TAIR 10 genome reference and gff3 annotation file were downloaded from phytozome v10 (phytozome.jgi.doe.gov/pz/portal.html). The maize B74 v4 genome reference and gff3 annotation file were downloaded from Ensembl Plants (http://plants.ensembl.org). Full-length high quality isoforms were mapped onto reference genomes using minimap2 version 2.16-r922 with options -x splice -uf --secondary=no -C5 (Li 2018). Cupcake ToFU collapse_isoforms_by_sam.py was used to combine isoforms mapping to an overlapping region of the genome without combining truncated 5’ end from different isoforms (option –dun-merge-5-shorter)

(https://github.com/Magdoll/cDNA_Cupcake/wiki). The number of full-length reads corresponding to each collapsed isoform was computed using home-made R scripts. For each gene of the genome reference, the gene Isoseq isoforms were identified as overlapping over at least 80% of the gene length or at least 80% of the Isoseq isoform length (results hold when using 50% overlap as a threshold, data not shown). Overlapping isoforms were then combined for each gene to compute the proportion of reads which transcription start site (TSS) is after the start of exon 1, causing aberrant transcription.

Analysis of RNA-seq data

A. thaliana RNA-seq reads were aligned to reference genome TAIR10 using Tophat v2.1.0 (Kim et al. 2013) supplied with a reference genome feature file (GFF) with the following arguments -I 50000 --b2-verysensitive --b2-D 50. We used raw counts generated by featureCounts (v1.6.4) (Liao et al. 2014) with options --fraction -p -O -f (paired reads were counted as one fragment and read count was divided by the number of exon to which the read or fragment mapped to). Genes with RPKM lower than one were discarded from statistical analyses.

Detection of intron retention

A. thaliana TAIR10 gff file was used to find intron only coordinates in the *A. thaliana* genome. Intron only coordinates were annotated as introns in at least one transcript and never annotated as untranslated terminal repeats (UTRs) nor exons in any other transcript. In Isoseq data, sense reads that mapped to at least part of an intron sequence were considered as having retained an intron. In RNA-seq data, we counted reads mapped to introns and normalized to obtain intron coverage in RPKM (reads per kilo base per million mapped reads).

Statistical study of the effects of gbM

The effect of gbM on aberrant transcription start site was studied using Isoseq data. Isoseq reads that started after the start of exon 1 were considered as aberrant TSS. For each gene, the proportion of conventional transcription start (i.e. the proportion of reads starting before the start of exon 1) was computed and analyzed in a generalized linear model (equation 6) using the R package lme4 (Bates et al. 2015) with the binomial family. In order to measure the effect of gene methylation state while controlling for differences in gene length and expression, three fixed effects were used (equation 6). Gene expression level was measured as the number of full-length Isoseq reads mapping to a gene. The Clontech SMARTer cDNA kit used for sequencing of the maize Isoseq data does not have a cap trap step. It is possible that full length reads come from 5’ degraded RNA. Indeed, it happens that 5’ transcripts ends (which correspond to the transcription start of the gene) undergo natural degradation in cells. Transcripts with degraded 3’ end are not selected in the sequencing process because only transcripts with poly-A tails are considered full-length. We hypothesize that the natural 5’ degradation will happen at the same rate for gbM genes and UM genes, so that we can still compare the proportion of aberrant TSS between gbM and UM genes. Another possible confounding factor is the inclusion of 5’ truncated transcripts in Isoseq data due to incomplete reverse transcription during sequencing. This bias affects both the maize and *A. thaliana* Isoseq datasets that we used here since it happens after transcript selection. We hypothesize that this bias will affect gbM and UM genes at the same rate. However, we should keep in mind that this effect of incomplete reverse transcription might depend on transcript length. We therefore included gene length in our model.

proportion of conventional TSS ~ gene methylation state + gene length + gene expression (6)

A similar approach was used to study the proportion of Isoseq reads with sens transcription and intron retention:

proportion of sens reads ~ gene methylation state + gene length + gene expression (7)

proportion of reads with intron retention ~ gene methylation state + gene length + gene expression (8)

References

Bates D, Mächler M, Bolker B, Walker S. 2015. Fitting Linear Mixed-Effects Models Using lme4. Journal of Statistical Software. doi: 10.18637/jss.v067.i01.

Bewick AJ et al. 2016. On the origin and evolutionary consequences of gene body DNA methylation. Proc. Natl. Acad. Sci. U.S.A. 113:9111–9116. doi: 10.1073/pnas.1604666113.

Cartolano M, Huettel B, Hartwig B, Reinhardt R, Schneeberger K. 2016. cDNA Library Enrichment of Full Length Transcripts for SMRT Long Read Sequencing. PLoS ONE. 11:e0157779. doi: 10.1371/journal.pone.0157779.

Kim D et al. 2013. TopHat2: accurate alignment of transcriptomes in the presence of insertions, deletions and gene fusions. Genome Biol. 14:R36. doi: 10.1186/gb-2013-14-4-r36.

Liao Y, Smyth GK, Shi W. 2014. featureCounts: an efficient general purpose program for assigning sequence reads to genomic features. Bioinformatics. 30:923–930. doi: 10.1093/bioinformatics/btt656.

Muyle A, Ross-Ibarra J, Seymour DK, Gaut BS. 2021. Gene body methylation is under selection in Arabidopsis thaliana. Genetics. 218:iyab061. doi: 10.1093/genetics/iyab061.

Seymour DK, Gaut BS. 2019. Phylogenetic shifts in gene body methylation correlate with gene expression and reflect trait conservation. Mol. Biol. Evol. doi: 10.1093/molbev/msz195.

Wang B et al. 2016. Unveiling the complexity of the maize transcriptome by single-molecule long-read sequencing. Nat Commun. 7:11708. doi: 10.1038/ncomms11708.

Zhang R et al. 2017. A high quality Arabidopsis transcriptome for accurate transcript-level analysis of alternative splicing. Nucleic Acids Res. 45:5061–5073. doi: 10.1093/nar/gkx267.

	Estimate	Standard Error	z-value	p-value
UM - gbM	0.214	0.0064	33.4	$2.52x10^{-16}$
Gene length	-0.178	0.0014	-130.7	$< 2x10^{-16}$
Gene expression	0.042	0.00061	69.15	$< 2x10^{-16}$

Supplementary Table S1: Estimation of the effect of gene methylation, gene length and gene expression on the proportion of conventional transcription start site in Isoseq data of **maize** using a generalized linear model (see Materials and Methods, equation 6). Unmethylated (UM) genes have significantly more conventional TSS. Longer genes have significantly fewer conventional TSS and genes with higher expression levels have significantly more conventional TSS.

	Estimate	Standard Error	z-value	p-value
gbM - UM	0.446	0.0309	131.05	$< 2x10^{-16}$
Gene length	-1.01	0.0151	-66.53	$< 2x10^{-16}$
Gene expression	0.176	0.00610	28.88	$< 2x10^{-16}$

Supplementary Table S2: Estimation of the effect of gene methylation, gene length and gene expression on the proportion of conventional transcription start site in Isoseq data of ***A. thaliana*** using a generalized linear model (see Materials and Methods, equation 6). gbM genes have significantly more conventional TSS. Longer genes have significantly fewer conventional TSS and genes with higher expression levels have significantly more conventional TSS.

	Estimate	Standard Error	z-value	p-value
–gbM - UM	2.01	0.108	18.52	$< 2x10^{-16}$
Gene length	NS	NS	NS	NS
Gene expression	16.23	0.417	38.94	$< 2x10^{-16}$

Supplementary Table S3: Estimation of the effect of gene methylation, gene length and gene expression on the proportion of sens Isoseq reads in ***A. thaliana*** using a generalized linear model (see Materials and Methods, equation 7). gbM genes have significantly more sens transcription. Gene length has no significant effect (NS) and genes with higher expression levels have significantly more sens transcription.

	Estimate	Standard Error	z-value	p-value
gbM - UM	1.04	0.16	65.1	$< 2x10^{-16}$
Gene length	0.43	0.0089	48.2	$< 2x10^{-16}$
Gene expression	16.23	0.417	38.94	$< 2x10^{-16}$

Supplementary Table S4: Estimation of the effect of gene methylation, gene length and gene expression on the proportion of sens Isoseq reads in **maize** using a generalized linear model (see Materials and Methods, equation 7). gbM genes have significantly less sense transcription. Longer genes and genes with higher expression levels have significantly more sense transcription.

	Estimate	Standard Error	z-value	p-value
–gbM - UM	0.212	0.0178	11,94	$2.26x10^{-10}$
Gene length	0.52	0.0104	50.13	$< 2x10^{-16}$
Gene expression	$2.96x10^{-4}$	$6.35x10^{-6}$	46.61	$< 2x10^{-16}$

Supplementary Table S5: Estimation of the effect of gene methylation, gene length and gene expression on the proportion of Isoseq reads that retain at least one intron in ***A. thaliana*** using a generalized linear model (see Materials and Methods, equation 8). UM genes have significantly less intron retention. Longer genes have significantly more intron retention and genes with higher expression levels have significantly more intron retention.
